# Numerical Evaluation of the Effects of Transducer Displacement on Transcranial Focused Ultrasound in the Rat Brain

**DOI:** 10.3390/brainsci12020216

**Published:** 2022-02-04

**Authors:** Hyeon Seo, Hyungkyu Huh, Eun-Hee Lee, Juyoung Park

**Affiliations:** 1 Medical Device Development Center, Daegu-Gyeongbuk Medical Innovation Foundation, Daegu 41061, Korea; hseo0612@gmail.com (H.S.); hkhuh@kmedihub.re.kr (H.H.); ehlee@kmedihub.re.kr (E.-H.L.); 2 Department of High-Tech Medical Device, College of Future Industry, Gachon University, Seongnam-si 13120, Korea

**Keywords:** transcranial focused ultrasound, acoustic simulation, single-element transducer, displacement

## Abstract

Focused ultrasound is a promising therapeutic technique, as it involves the focusing of an ultrasonic beam with sufficient acoustic energy into a target brain region with high precision. Low-intensity ultrasound transmission by a single-element transducer is mostly established for neuromodulation applications and blood–brain barrier disruption for drug delivery. However, transducer positioning errors can occur without fine control over the sonication, which can affect repeatability and lead to reliability problems. The objective of this study was to determine whether the target brain region would be stable under small displacement (0.5 mm) of the transducer based on numerical simulations. Computed-tomography-derived three-dimensional models of a rat head were constructed to investigate the effects of transducer displacement in the caudate putamen (CP) and thalamus (TH). Using three different frequencies (1.1, 0.69, and 0.25 MHz), the transducer was displaced by 0.5 mm in each of the following six directions: superior, interior, anterior, posterior, left, and right. The maximum value of the intracranial pressure field was calculated, and the targeting errors were determined by the full-width-at-half-maximum (FWHM) overlap between the free water space (FWHM_water_) and transcranial transmission (FWHM_base_). When the transducer was positioned directly above the target region, a clear distinction between the target regions was observed, resulting in 88.3%, 81.5%, and 84.5% FWHM_water_ for the CP and 65.6%, 76.3%, and 64.4% FWHM_water_ for the TH at 1.1, 0.69, and 0.25 MHz, respectively. Small transducer displacements induced both enhancement and reduction of the peak pressure and targeting errors, compared with when the transducer was displaced in water. Small transducer displacement to the left resulted in the lowest stability, with 34.8% and 55.0% targeting accuracy (FWHM_water_) at 1.1 and 0.69 MHz in the TH, respectively. In addition, the maximum pressure was reduced by up to 11% by the transducer displacement. This work provides the targeting errors induced by transducer displacements through a preclinical study and recommends that attention be paid to determining the initial sonication foci in the transverse plane in the cases of small animals.

## 1. Introduction

Focused ultrasound (FUS) is a promising therapeutic technique with applications in various areas, such as tumor ablation, drug delivery, and neuromodulation, because it enables noninvasive, incision-free transcranial treatment by delivering acoustic energy through the skull. The use of high-intensity FUS (HIFU) for targeted tissue ablation has been investigated extensively, and clinical efficacy has been achieved in the treatment of essential tremors [1,2,3,4]. To realize a precise focus on a thermal coagulative lesion in the brain without damaging the intervening tissue, magnetic resonance (MR)-guided FUS (MRgFUS) systems have been used with multi-element phased-array transducers. MRgFUS with a multi-element transducer can accurately and transiently target brain regions with real-time MR guidance by rectifying phase aberrations by relying on prior knowledge of the skull geometry and simultaneous MR thermometry [4,5,6,7,8].

Moderate levels of nonthermal, pulsed ultrasound waves can be employed for neuromodulation and the microbubble-medicated disruption of the blood–brain barrier (BBB) to facilitate drug delivery to the brain. Significant interest has developed in the application of low-intensity FUS for neuromodulation, owing to the ability of ultrasound to penetrate deep brain regions at high spatial resolution. Numerous experiments with different sonication parameters (frequency, pressure amplitude, duration, and duty cycle) have demonstrated both excitatory and inhibitory neuromodulatory effects on animal models and humans [9,10,11,12]. In addition, FUS with microbubbles resulted in stable microbubble oscillations within the brain microvessels that demonstrated a temporary increase in the permeability of the BBB [13]. The localized acoustic focus prior to FUS procedures can be directly visualized using MR acoustic radiation force imaging [14]. For FUS-induced BBB disruption, recent clinical studies [15,16,17] have been conducted with the following ongoing ClinicalTrials.gov identifiers: NCT03321487, NCT02343991, NCT03608553, NCT03616860, and NCT03626896.

In contrast to HIFU systems with multiple arrays, which have limited availability, owing to their high cost and complexity, a single-element focused transducer has been used in the majority of research on FUS-induced neuromodulation and BBB disruption, with a narrow band of ultrasound power applied over a target area [18,19,20,21,22]. A single-element focused transducer can also deliver focused acoustic energy to the brain with a high degree of precision (on the order of several millimeters). However, individual variation has been reported in tactile sensations, which may be due to individual variations in local neuroanatomy, head motion, or acoustic field translation [18]. When the spatial specificity of FUS was tested according to the transducer translation, this displacement yielded an abolished modulatory effect based on electrophysiological observations and targeted spatially discrete brain regions [19].

In research on FUS-induced BBB disruption, a single-element focused transducer combined with an MRgFUS system can be used to monitor the degree of BBB disruption after FUS with a contrast-enhanced MR signal. Instead of developing a combined MRgFUS system, neuronavigation systems have been developed, resulting in a simple and efficient targeting procedure for sonication that does not require an MRI system [22,23,24,25]. In both MRgFUS and neuronavigation systems, targeting errors could be caused by displacement between the subject and transducer or system registration errors. Furthermore, a single-element transducer must be physically moved to aim at the target, and this initial sonication focus is identified manually using MR images. Therefore, it is possible to displace the transducer, which can induce potential errors unless care is taken [26]. These positioning errors will affect repeatability and cause reliability problems irrespective of whether the externally applied ultrasound is transmitted accurately and sufficient pressure is delivered to the target region.

Many FUS technologies have been developed for clinical applications; however, they are still in the preclinical stages for the optimization and assessment of the underlying mechanisms and biological events induced by FUS treatment [26,27]. Furthermore, the small sizes of rodents, such as rats and mice, make FUS experiments difficult because the ultrasound beam can be comparable to the target size; thus, high precision is required for localization to avoid exposing the surrounding tissue [28].

The numerical simulations of transcranial propagation are used to predict the intracranial field because the skull causes a significant distortion of the transmitted ultrasound beam. To ensure accuracy in the numerical simulations, a sensitivity examination of the intracranial field was performed to determine the impact of the geometry and acoustic properties of the model [29,30,31]. Park et al. proposed a method to find the optimal position of a single-element transducer [32], and Kim et al. designed a dual-crossed transducer system to achieve high spatial resolution for small animals [33]; however, even though we found the optimal position of the transducer through the numerical simulation, targeting errors could be caused by the registration process between the simulation and experiments. As for numerical studies performed with rodents, Constans et al. simulated the pressure field and thermal rise under a large range of frequencies for the specific target area. They found the prominent impact of standing waves at the lowest frequencies [34,35]. Until now, numerical simulation studies have shown that small variations in the geometry and acoustic properties of the model could make significant impacts on the focus location or spatial dimension.

The objective of this work was to investigate, numerically, the effects of potential errors due to transducer displacement on transcranial ultrasonic beams using a single-element focused transducer in a rat brain. We considered the effects of a small (0.5 mm) transducer displacement to evaluate whether the stimulation of a defined brain region would be stable under small displacements, because FUS shows a high degree of precision on the order of several millimeters. We developed three-dimensional computational models of a rat and single-element transducer at 1.1 MHz to target the caudate putamen (CP) and thalamus (TH). The effects of the acoustic medium properties of the skull and brain were examined. By comparing different acoustic models, an acoustic model of the skull with homogeneous properties was used to evaluate whether the distortion due to the rat skull was minor. Then, we simulated the defined brain regions being targeted stably with small transducer displacements using three different frequencies (1.1, 0.69, and 0.25 MHz). Three-dimensional simulations were performed, and the FUS-induced pressure field between the free water space and intracranial space was evaluated to assess the targeting accuracy.

## 2. Materials and Methods

### 2.1. Ultrasound Propagation Model

To simulate the pressure-field distributions induced by FUS, we constructed a three-dimensional acoustic model of a rat (male Sprague Dawley rat; eight weeks old; weighing 370 g), including the skull, cerebrospinal fluid (CSF), and brain, using microcomputed tomography (R_mCT2, Rigaku, Japan) images. The image size was 512 × 512 × 512 pixels, with a cubic voxel size of 0.08 mm. The images were resampled with linear interpolation at 0.17 mm to reduce the computational time, and the pixel size was smaller than seven points per wavelength. The skull layer was segmented using the open-source application Seg3d, which offers an interactive segmentation function for image data [36] that was employed to construct the acoustic model of the skull. We utilized the Otsu threshold filter to produce the skull mask, and it was smoothed by running the “smooth dilate-erode” tool. The brain was formed artificially by shrinking the inner surface of the skull by 0.15 mm [37]. A linear simulation was performed based on the assumption that the impact of shear wave propagation is nominal [38,39]. Simulations were conducted using the open-source k-Wave MATLAB toolbox [40], which solves the acoustic equations on a spatial grid by utilizing a k-space pseudospectral scheme.

Three-dimensional maps of the skull, brain, and tissues were extracted from CT images, and the brain was assumed to be a homogeneous medium [32,41]. To reduce the model complexity and computation time, the simulation domain excluding the skull layer can be assumed to be water to reduce the model complexity and computational time [31,35]. To investigate the effects of the acoustic medium properties of the rat skull and brain, we assigned three different acoustic models: (1) the skull had homogenous properties, and the CSF and brain were assumed to have the same acoustic properties as water (homogeneous skull model); (2) the skull, CSF, and brain had different homogeneous properties (homogeneous three-layered (3L) model); and (3) the skull had heterogeneous properties, which considered the effects of the porosity of the skull on wave propagation, and the CSF and brain had the same acoustic properties as water (heterogeneous skull model). Table 1 summarizes the acoustic properties of the skull, brain, and water. For heterogeneous properties, the acoustic properties of the speed of sound (c), density (ρ), and attenuation (α) were assumed to be proportional to the normalized CT Hounsfield units (0≤φ≤1), as follows [7]:

(1) $$c=c_{\mathrm{water}}\varphi +c_{\mathrm{skull},\mathrm{hetero}}\left(1-\varphi \right),$$

(2) $$\rho =\rho_{\mathrm{water}}\varphi +\rho_{\mathrm{skull},\mathrm{hetero}}\left(1-\varphi \right),$$

(3) $$\alpha =\alpha_{\min,\mathrm{skull},\mathrm{hetero}}+\left(\alpha_{\max,\mathrm{skull},\mathrm{hetero}}-\alpha_{\min,\mathrm{skull},\mathrm{hetero}}\right)\varphi^{0.5}.$$

The bowl-shaped transducer model was a spherically curved single-element FUS transducer (FUS Instrument, Toronto, ON, Canada) with a diameter of 75 mm, focal length of 60 mm, and center frequency of 1.1 MHz. The acoustic model was coupled with the ultrasound source by considering the space between the model and transducer as water. The power of the transducer was set to obtain a maximum pressure of 0.65 MPa at focus in free water at 1.1 MHz [42].

**Table 1:** Acoustic parameters [43,44,45,46,47].

Speed of Sound (m s^−1^)	Density (kg m^−3^)	Attenuation (dB MHz^−1.43^ cm^−1^)
$c_{\mathrm{water}}=1482$	$\rho_{\mathrm{water}}=1000$	$\alpha_{\mathrm{water}}=0.24\times 10^{-2}$
$c_{\mathrm{skull},\;\mathrm{homo}}=2850$	$\rho_{\mathrm{skull},\mathrm{homo}}=1732$	$\alpha_{\mathrm{skull},\mathrm{homo}}=8.83$
$c_{\mathrm{skull},\mathrm{hetero}}=3100$	$\rho_{\mathrm{skull},\mathrm{hetero}}=2200$	$\alpha_{\min,\mathrm{skull},\mathrm{hetero}}=12.67$, $\alpha_{\max,\mathrm{skull},\mathrm{hetero}}=51.42$
$c_{\mathrm{brain}}=1545$	$\rho_{\mathrm{brain}}=1030$	$\alpha_{\mathrm{brain}}=6.9\times 10^{-2}$

For all the simulations, a linear acoustic simulation was performed. The default Courant–Friedrichs–Lewy stability criterion (0.3) and a sonication pulse duration of 103 μs (default simulation k-wave length) were applied. The simulation domains for the acoustic model with the transducer included 530 × 559 × 530 grid points, and each 3D simulation required a computer node with a 16-core AMD Ryzen 2950X 3.50 GHz central processing unit and 128 GB of random-access memory and approximately 35 h to solve.

### 2.2. Transducer Displacements and Target Brain Regions

**Figure 1:**
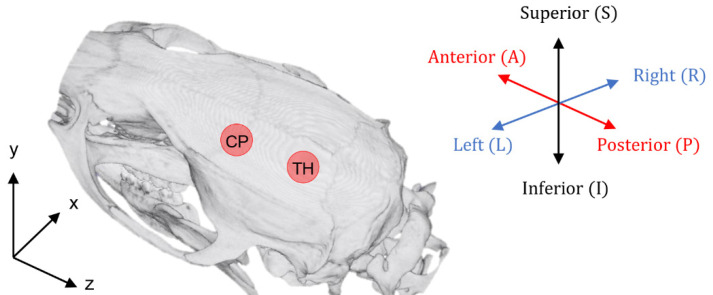
Skull model indicating FUS targets and the process of displacement by 0.5 mm in any of six directions—+S, +I, +A, +P, +L, or +R—relative to its base location from the CP and TH.

When we tested the transducer displacement, we used the homogeneous skull model and investigated various frequencies (1.1, 0.69, and 0.25 MHz) with identical parameters. The frequencies of 1.1, 0.69, and 0.25 MHz were chosen by considering previous studies regarding FUS-induced BBB disruption [42,48,49,50,51,52]. The transducer placement was determined in accordance with previous experimental conditions to achieve FUS-BBB disruption [48]. As shown in Figure 1, the base positions of the transducer in CP and TH were determined relative to the bregma, as follows (in mm): AP: +0.0, ML: +2.0 for CP; AP: −5.5, ML: +2.0 for TH. For each target, the acoustic model was simulated seven times, wherein the transducer was firstly placed at the base target position and was then displaced by 0.5 mm in one of the six directions: superior (+S), interior (+I), anterior (+A), posterior (+P), right (+R), or left (+L) (Figure 1).

### 2.3. Analysis

Differences in the intracranial fields due to the transducer displacement were quantified through the peak focal pressure and the volume of focus (calculated using the full-width-at-half-maximum volume with a pressure amplitude above 50% of the peak pressure). The targeting errors as a function of the transducer displacement were quantified using the FWHM volume overlap between free space (the ideal model) and after transcranial transmission (the simulation model), denoted as FWHM_water_. The ideal model was simulated by assigning homogeneous water properties (Table 1), which indicated the acoustic pressure in a free water domain. The simulated acoustic model was subjected to a displacement test that considered the acoustic properties of the skull. We then quantified the volume associated with the FWHM of the pressure, and the FWHM overlap was assessed to determine which part of the FWHM volume in the simulation model overlapped with the FWHM volume in the ideal model. FWHM_water_ estimated the percentage of the FWHM volume in the simulation models that was inside the FWHM volume in the free water space. The percentage ranged from 0% (the focus of acoustic pressure occurred outside the target region, indicating large distortion) to 100% (there was no distortion due to the skull, and thus, acoustic pressure was delivered to the target region perfectly). We also calculated FWHM_base_, which indicated the FWHM overlap between the intracranial pressure field at the base position (the base model) and after transducer displacement, because the FWHM volume as a function of the transducer displacement may have been equal to that of the base model.

## 3. Results

### 3.1. Model Validation

**Figure 2:**
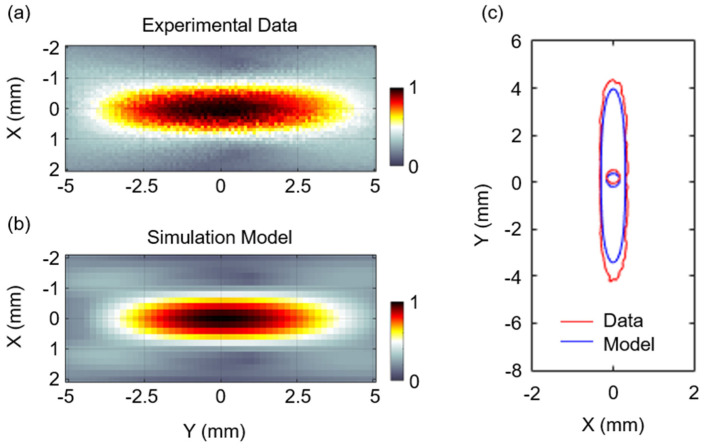
Normalized acoustic pressure distribution in a free water domain induced by FUS at 1.1 MHz in the (**a**) experiment and (**b**) simulation. The pressure−field distributions are centered at the peak points. (**c**) Comparison of the experimental and simulated data using FWHM contours and markers at the centroids of the contours.

To validate the FUS simulation, we conducted both a numerical simulation and in vitro measurements of the acoustic field in free water space. The free-field ultrasound pressure was measured with an acoustic intensity measurement system (AIMS III, ONDA, Sunnyvale, CA, USA) with a hydrophone (HGL-400, ONDA, Sunnyvale, CA, USA). A single-element, spherically curved piezoelectric transducer (FUS Instrument, Toronto, Canada) with a center frequency of 1.1 MHz was used with the same configuration as the transducer model. The peak pressure (MPa) centered at the focus point was measured at a spatial resolution of 100 μm and was normalized by the peak pressure in water. We then constructed a 3D acoustic model to recreate the empirical acoustic pressure in free water space and assigned homogeneous water properties, as listed in Table 1. The peak pressure field was compared between the in vitro pressure measurements and numerical results to estimate the simulation accuracy (Figure 2). The focus was 1.59 mm in diameter and 8.48 mm in length along the sonication axis at the FWHM of the acoustic pressure map in the experiments. The numerical results yielded agreed well, quantitatively, with the empirical observations, which resulted in comparable FWHM profiles (1.51 mm in diameter and 7.41 mm in length).

### 3.2. Effects of Acoustic Properties

**Figure 3:**
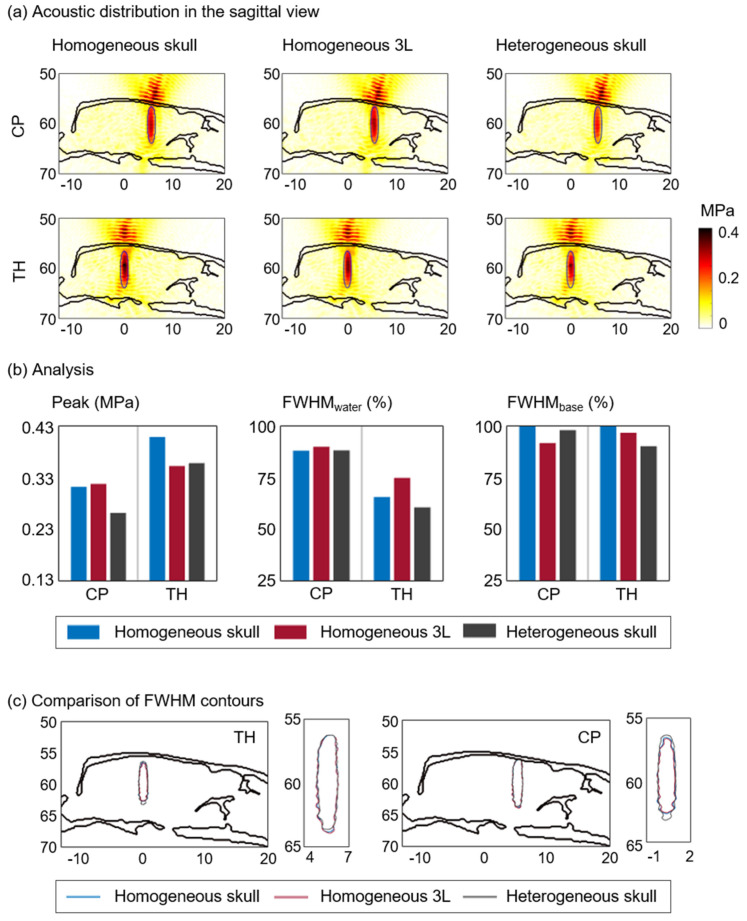
(**a**) Simulated acoustic pressure distributions and (**b**) analysis of the distributions corresponding to the homogeneous skull, homogeneous 3L, and heterogeneous skull models at 1.1 MHz. The intracranial maximum pressure, FWHM_water_, and FWHM_base_ are compared. (**c**) FWHM contours of the homogeneous skull (blue line), homogeneous 3L (red line), and heterogeneous skull (black line) models.

We constructed three different acoustic models, in which the 3D skull model was assumed to be homogeneous (homogeneous skull model) or heterogeneous (heterogeneous skull model), as well as a homogeneous 3L model consisting of the skull, CSF, and brain with different homogeneous properties. At 1.1 MHz, we compared three different acoustic models (homogeneous skull, homogeneous 3L, and heterogeneous skull models) to investigate the effects of the detailed acoustic properties of the rat skull and brain on the intracranial pressure field. Figure 3 shows the acoustic pressure distributions in the sagittal view and their analyses.

Although the acoustic distortion is known to be minor, owing to the thin skulls of rodents, we found 2.10-, 2.06-, and 2.50-fold decreases in maximum pressure for the CP and 1.60-, 1.86-, and 1.83-fold decreases in maximum pressure for the TH with the homogeneous skull, homogeneous 3L, and heterogeneous skull models, respectively. For the FWHM overlap with respect to free water, we found values of 88.3%, 90.1%, and 88.4% for FWHM_water_ for the CP and 65.7%, 75.1%, and 60.6% for FWHM_water_ for the TH with the homogeneous skull, homogeneous 3L, and heterogeneous skull models, respectively. When we set the homogeneous skull model as the base model and calculated FWHM_base_, we found that both the homogeneous 3L and heterogeneous skull models had FWHM_base_ > 90%. Accordingly, we found similarly affected volumes between the homogeneous skull, homogeneous 3L, and heterogeneous skull models, which are represented by the FWHM contours (Figure 3c). Therefore, the homogeneous skull model that assigned constant acoustic properties was sufficient for calculating the intracranial pressure field for the transducer displacement tests because this model reduced the model complexity and computation time.

### 3.3. Effects of Transducer Displacements

**Figure 4:**
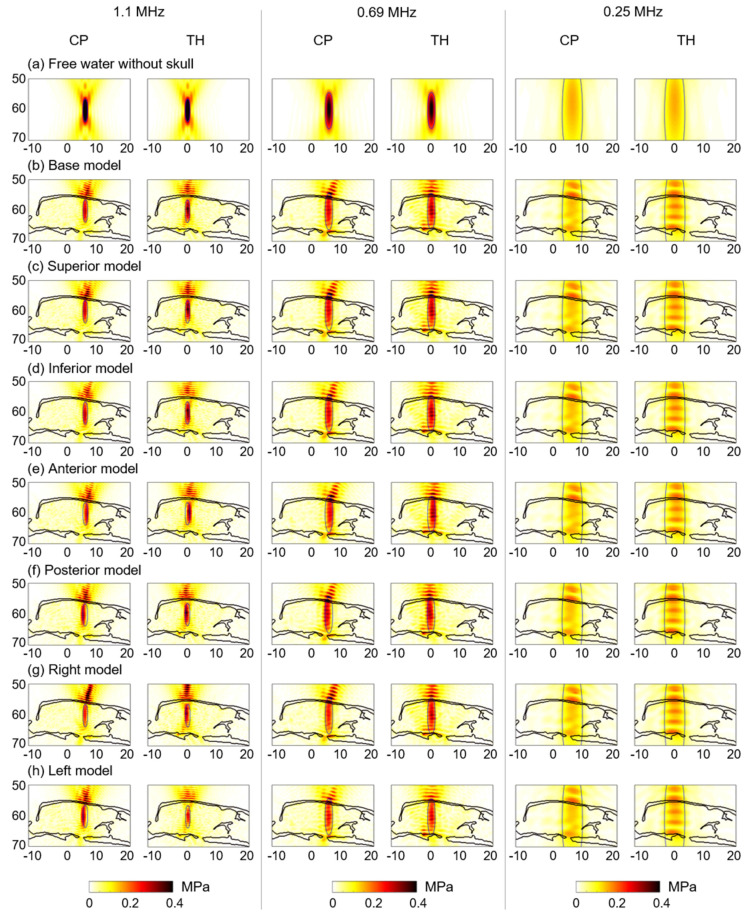
Acoustic distributions at 1.1, 0.69, and 0.25 MHz in the sagittal view (**a**) in free water and (**b**) after transcranial transmission for the CP and TH. The transducer is displaced by 0.5 mm in all six directions—(**c**) +S, (**d**) +I, (**e**) +A, (**f**) +P, (**g**) +R, and (**h**) +L—relative to its base location. The blue lines denote the half−maximum pressure profiles in free water space.

For the transducer displacement test, the 3D model of the rat skull was assumed to have homogeneous acoustic properties, and various frequencies (1.1, 0.69, and 0.25 MHz) were considered. Figure 4 shows the acoustic pressure field as a function of the transducer displacement at all frequencies. In free water (Figure 4a), the brain targets exhibit increased volumes (FWHM volumes) and the pressure field is reduced with decreasing frequency. After transcranial transmission (Figure 4b–h), there are more prominent standing waves at 0.25 MHz than at the other frequencies. The pressure-field distributions normalized to peak acoustic pressure showed variations in focal volume size and interference patterns as a function of input frequency (Supplementary Figure S1).

**Figure 5:**
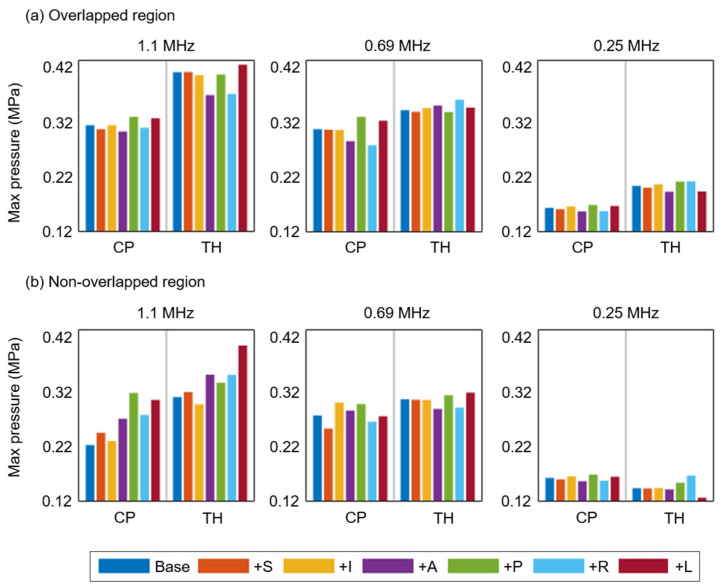
Effects of transducer displacement on intracranial maximum pressure in the overlapped (**a**) and non-overlapped (**b**) focal volumes compared with the base model at 1.1, 0.69, and 0.25 MHz. The transducer was displaced by 0.5 mm in all six directions—+S, +I, +A, +P, +L, and +R—relative to its base location.

When we analyzed the intracranial maximum pressure in the overlapped (Figure 5a) and non-overlapped (Figure 5b) focal volumes compared with the base model. All the maximum pressure values were observed in the overlapped focal volumes under the transducer displacements. For the intracranial maximum pressure in the overlapped focal volume, we found that the maximum pressures were generally lower in the CP and higher in the TH. After transducer displacement, we observed the largest reduction in the maximum pressure when the transducer moved in the +A direction at frequencies of 1.1 MHz (3.7% for the CP; 11.4% for the TH) and 0.25 MHz (4.2% for the CP; 5.6% for the TH). At 0.69 MHz, the +R and +P displacements induced the largest reduction in the maximum pressure in the cases of the CP (10.6%) and TH (1.0%), respectively. In general, +P movement yielded a higher pressure than +A movement, except in the case of the TH at 0.69 MHz. For the intracranial maximum pressure in the non-overlapped focal volume (Figure 5b), we found that the maximum pressure value was generally higher when the transducer moved in the transverse plane, indicating reduced targeting accuracy at a center frequency of 1.1 MHz. When we analyzed the normalized intracranial maximum pressure, larger variations were clearly observed when the transducer was displaced in the transverse plane at 1.1 MHz (Supplementary Figure S2).

**Figure 6:**
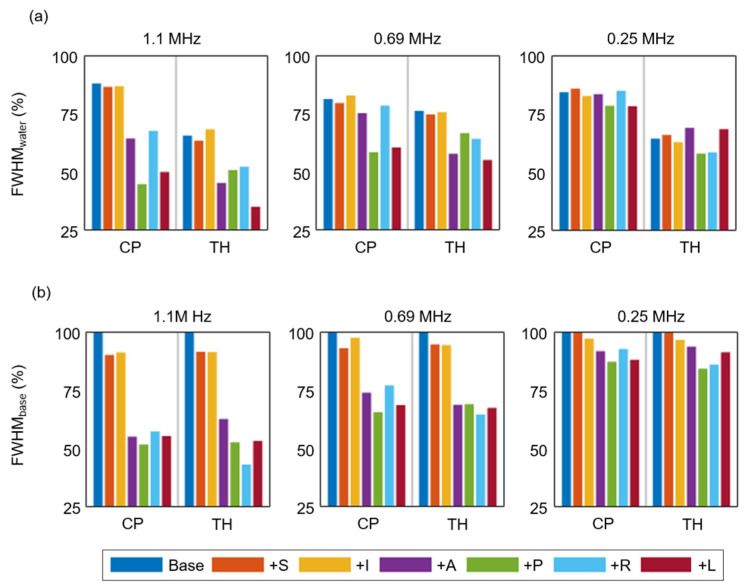
Effects of transducer displacement on (**a**) FWHM_water_ and (**b**) FWHM_base_ at 1.1, 0.69, and 0.25 MHz. The transducer was displaced by 0.5 mm in all six directions—+S, +I, +A, +P, +L, and +R—relative to its base location.

Clear distinctions between the target regions at the base target positions were observed in FWHM_water_ (Figure 6a), which resulted in a lower targeting accuracy in the TH (1.1 MHz: 65.7%; 0.69 MHz: 76.3%; 0.25 MHz: 64.4%) than in the CP (1.1 MHz: 88.3%; 0.69 MHz: 81.5%; 0.25 MHz: 84.5%). In general, the transducer displacement in the transverse plane (+A, +P, +R, and +L) showed a relatively significant decrease in FWHM_water_ compared with the case in which we adjusted the distance between the transducer and target (+S and +I directions). In particular, a small displacement of the transducer in the +L direction resulted in the lowest stability, with 34.8% and 55.0% targeting accuracy (FWHM_water_) at 1.1 and 0.69 MHz in the TH, respectively. These transducer displacement effects were reduced by lowering the frequency from 1.1 MHz to 0.25 MHz. Accordingly, we found more significant decreases in FWHM_base_ with transverse plane displacements than with +S and +I displacements (Figure 6b). At 1.1 MHz, the transducer displacements in the transverse plane showed values of around 50% for FWHM_water_ and FWHM_base_, indicating that only half of the FWHM volume fell into the target we intended.

**Figure 7:**
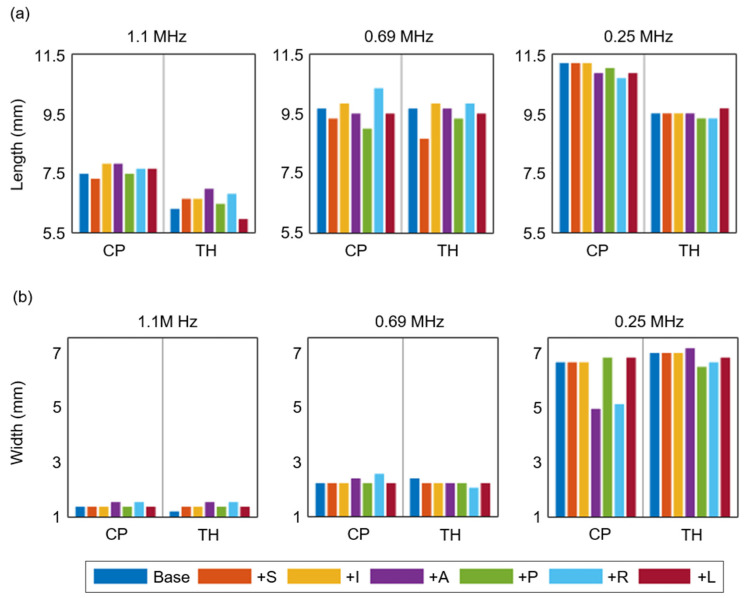
The comparisons of length (**a**) and width (**b**) of FWHM profiles at 1.1, 0.69, and 0.25 MHz under the transducer displacements in all six directions relative to its base location.

The FWHM profiles were further analyzed by the focal dimensions of length and width (Figure 7), and we found variations in the lengths of the focal volumes for different target locations. At 1.1 MHz, the focal volume at the CP showed dimensions comparable to those of the free water case (7.39 mm in length and 1.51 mm in width), and the focal volume at TH was reduced compared to that in the CP. At 0.25 MHz, we found a consistently shorter length in the TH compared to that in the CP because of interference depending on the geometry. According to the transducer displacements, the focal dimension was varied, which indicates the importance of geometry.

## 4. Discussion

In this study, we investigated the effects of transducer displacements on the targeting accuracy in a rat brain via the numerical simulation of the acoustic pressure field. Before performing the displacement test, the effects of the acoustic properties of the model were examined by comparing homogeneous, homogeneous 3L, and heterogeneous skull models. We obtained comparable FWHM contours using all three models, indicating that the skull geometry is more influential than the skull porosity or soft tissues in rat acoustic modeling. The transducer displacement induced both enhancements and reductions of the intracranial pressure field, and a distinction between target volumes was observed that generally resulted in a higher pressure in the TH than in the CP. Targeting accuracy assessments according to the transducer displacement were conducted based on the FWHM overlap, and we found a distinction between target regions that resulted in low targeting accuracy in the TH compared to the CP. Displacements of the distance between the transducer and targets (+S and +I movements) had minor impacts on the targeting accuracy, with values of 91.3%, 95.2%, and 98.6% for FWHM_base_ (overall average based on the CP and TH) at 1.1, 0.69, and 0.25 MHz, respectively. In contrast, transducer displacements in the transverse plane (+A, +P, +R, and +L) yielded values of 53.8%, 69.4%, and 89.6% for FWHM_base_ (overall average based on the CP and TH) at 1.1, 0.69, and 0.25 MHz, respectively. Although we found comparable targeting errors on average compared with when the transducer was displaced in the transverse plane in free water (1.1 MHz: 51.5%; 0.69 MHz: 70.5%; and 0.25 MHz: 87.3%), the FWHM_water_ was as low as 35% at 1.1 MHz, 55% at 0.69 MHz, and 58% at 0.25 MHz in some cases. These findings highlight the importance of ensuring accurate transducer placement in the transverse plane to convey sufficient acoustic energy to a target brain region and avoid exposing the surrounding tissues.

It was found that the affected area (FWHM volume) was larger with reduced pressure gain when the frequency was lower (Figure 4a). At 0.25 MHz, considerable interference patterns were observed because the skull was substantially smaller than the affected area (FWHM volume), and the interaction between the acoustic beam and skull was increased. In addition, the intracranial pressure was higher in the base model (CP: 0.16 MPa and TH: 0.20 MPa) than in free water (0.15 MPa). These findings are in agreement with previous numerical simulations, which revealed substantial intracranial pressure increases with interference patterns in rat skulls compared to the case in free water at low frequencies [34,53]. Owing to the large focal volume at 0.25 MHz, we found that the effect of the transducer displacement on the targeting accuracy was minor.

The single-element transducer was placed at a normal incidence angle to deliver higher acoustic pressure to the focal location effectively [32,48,54,55]. A recent study by Huh et al. demonstrated the effect of the incidence angle on the BBB permeability using a 1.1 MHz single focused transducer in terms of K_trans_, which was evaluated by performing dynamic contrast-enhanced MRI [48]. K_trans_ exhibited a negative correlation with the incidence angle (R2 = 0.7972) and a positive correlation with the simulated peak pressure (R2 = 0.4152). Following the study by Huh et al., we considered the relation between the maximum pressure and incidence angle, except in the cases of +S and +I displacements. The 3D incident angle was calculated using average normal vectors on the skull surface within the ultrasound focal area (1 mm^2^). In addition, we calculated the correlation between the maximum pressure and skull thickness (Table 2). In accordance with the earlier study, we found the impact of the incidence angle on the pressure field, which resulted in negative correlations between the maximum pressure and incidence angle at all frequencies (R2 = 0.7434 for 1.1 MHz, R2 = 0.5830 for 0.69 MHz, and R2 = 0.9143 for 0.25 MHz). In the base model, the targets in the TH yielded a higher pressure with a lower incidence angle (7.9°) compared with those in the CP (12.4°). No relationship was found between the maximum pressure and skull thickness along the central axis of the transducer. The minor impact of the skull thickness on the pressure field may be due to the thinness (around 0.5 mm) of the rat skull.

The simulation validation was performed at a frequency of 1.1 MHz, corresponding to the calibration experiments performed in free water. However, to estimate the in situ pressure correctly, further experimental measurements using a rat skull inside a water tank should be performed. Cho et al. measured the ultrasound pressure distribution with and without a rat skullcap and found a peak pressure in the range of 0.3–0.35 MPa through the rat skullcap when a peak acoustic pressure range of 0.6–0.65 MPa in free water was applied using a 1 MHz transducer [42]. Like Cho et al., we found a peak pressure in the range of 0.30–0.33 MPa after transcranial transmission in the CP and a peak pressure of 0.65 MPa in free water at 1.1 MHz, both of which fell within the experimental range. Although comparison with previous experimental data is difficult due to the use of different acoustic parameters, numerical simulations in rat, monkey, and human skulls have been experimentally validated in previous studies [34,41,53,56]. Thus, even without considering the experimental validity with the rat skull, the acoustic model we used may be helpful for understanding the effects of transducer displacement.

Considerable advances have been documented regarding the delivery of highly focused acoustic energy via FUS (on the order of a few millimeters) across the brain, including in the deeper subcortical regions. Thus, one important nonbiological factor that contributes to FUS effects is the precise positioning of the transducer. Although a phased-array transducer operating in conjunction with an MRgFUS system enables multiple ultrasound waves to converge at a single focus accurately, multi-array transducers are very expensive to purchase, especially for preclinical research. Although multi-element phased-array transducers have been extensively investigated to correct beam aberrations for noninvasive ablation in the brain and to determine the phases and amplitudes of phased arrays via acoustic simulations with CT images [4,5,6,7,8], single-element transducers have been used by physically moving them to targets, which may induce targeting errors [26]. For example, according to the RK-100 FUS Research System (FUS Instruments, Toronto, Canada), which is a preclinical small MR/CT-guided FUS research device, the targets can be identified manually in MR images. Thus, MR coordinates can be input into image-guided sonication planning software to move the transducer accurately into the appropriate position for sonication. Therefore, positioning errors can occur without fine control over the sonication.

Neuronavigation, which is an alternative approach for MRgFUS, also yielded targeting errors on the order of a few millimeters [22,23,24]. In a neuronavigation system, transducer positioning errors can arise from several sources, such as the registration between the physical and virtual MRI spaces and fiducial localization errors; these may induce pressure field variations owing to the inhomogeneity of the skull. Therefore, investigation of how the transducer displacement affects the targeting accuracy is informative for the further interpretation of primary and secondary outcomes after FUS.

There are several limitations to this study. Here, we aimed to determine whether the targeted brain region would be stable under small displacements, and thus, we selected a transducer displacement of 0.5 mm. Within 0.5 mm displacement, we found significant alteration in the targeting accuracy at 1.1 MHz and 0.69 MHz. Owing to the larger FWHM volume at 0.25 MHz, we found minor differences, as expected. Although we considered small changes in the transducer position, further studies on increasing the transducer displacement would be useful to determine the acceptable physical positioning error for low-frequency FUS. Another limitation is that this study was performed with one transducer geometry in a single animal model. In order to provide a more generalized conclusion, computational models from more subjects may be necessary to further substantiate our results.

The current investigation was focused on modeling the effects of transducer displacements on the intracranial pressure field generated by FUS in a preclinical study, and we demonstrated that transverse transducer movement significantly alters the targeting accuracy. Previous studies have shown that the inhomogeneity of the human skull plays a major role in attenuation, diffraction, and the bending of the beam path [7,31,32,41,57]. Thus, this positioning error would increase in larger animals and human subjects. These results provide insights into the importance of careful and consistent transducer placement for both preclinical and clinical studies. In this study, the ultrasound propagation model was simulated in an entire rat head, and the resulting targeting errors may vary between animals [58,59]. It should be noted that previous work using a rat CT scan has revealed good agreement with experimental measurements [34,35], and the results of this study indicated that precise focal spot positioning is essential in a preclinical study. Further transducer position characterization according to the individual should be strongly encouraged for reproducibility between experiments. Therefore, in accordance with the neuronavigation system proposed by Wu et al., not only image-guided sonication but also numerical simulation integration is required to predict the pressure field within the brain [23]. This requirement exists because the heterogeneity of the skull bone causes the disruption and shifting of the focal location. More importantly, we recommend that special care be exercised in determining the initial sonication foci of small animals at high frequencies (0.69 and 1.1 MHz), which will make FUS experiments difficult, as high precision is required for the localization of ultrasound to the target area.

## Figures and Tables

**Table 2:** Angle and skull thickness as functions of transducer displacement.

		Base	+A	+P	+R	+L
Angle (°)	CP	12.42	12.32	12.35	11.99	13.31
	TH	7.96	8.20	7.36	7.05	8.45
Thickness (mm)	CP	0.50	0.54	0.51	0.54	0.52
	TH	0.51	0.50	0.53	0.55	0.50

## Data Availability

The authors confirm that the data supporting the findings of this study are available within the paper. Raw data that support the findings of this study are available from the corresponding author, upon reasonable request.

## Supplementary Materials

The following supporting information can be downloaded at: https://www.mdpi.com/article/10.3390/brainsci12020216/s1. Figure S1: Normalized acoustic distribution at 1.1, 0.69, and 0.25 MHz;Figure S2: Effects of transducer displacement on normalized intracranial maximum pressure.
