# Supplementary material for: Numerical Evaluation of the Effects of Transducer Displacement on Transcranial Focused Ultrasound in the Rat Brain
Source: Brain Sci. 2022 Feb 4;12(2):216. doi: 10.3390/brainsci12020216 (PMC8870101; doi:10.3390/brainsci12020216)
Supplement: Supplementary file 1 [file brainsci-12-00216-s001.zip › brainsci-1531105-supplementary.pdf]

Supplementary Figures

Numerical evaluation of the effects of transducer displacement on transcranial focused ultrasound in the rat brain

Hyeon seo[1], Hyungkyu Huh[1], Eun-Hee Lee[1], and Juyoung Park[1,2]*

[1] Medical Device Development Center, Daegu-Gyeongbuk Medical Innovation Foundation, Daegu 41061, South Korea
[2] Department of High-Tech Medical Device, College of Future Industry, Gachon University, Seongnam-si 13120, South Korea
* Correspondence: opedoors@gmail.com

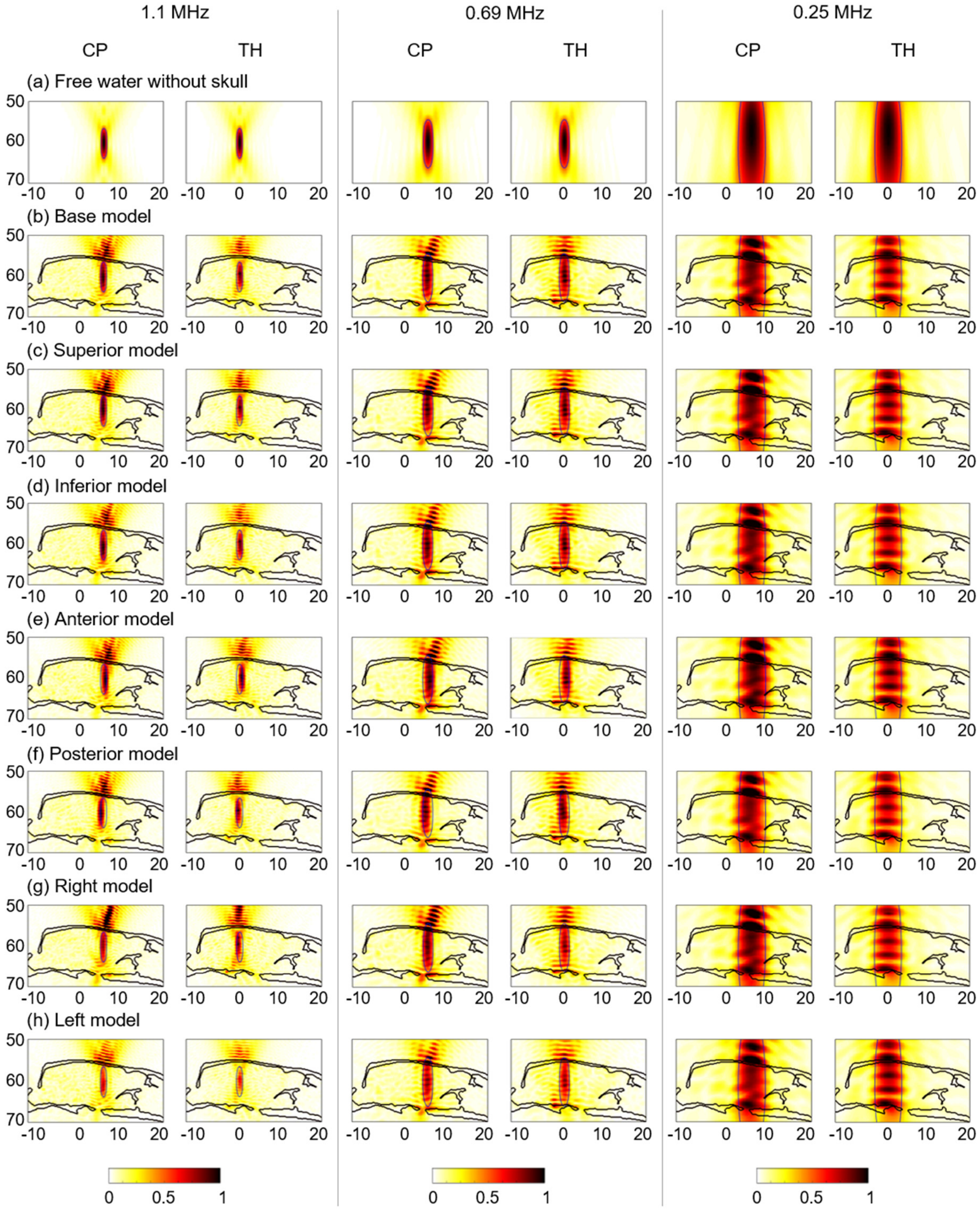

Supplementary Figure S1. Normalized acoustic distributions at 1.1, 0.69, and 0.25 MHz in the sagittal view (a) in free water and (b) after transcranial transmission for the CP and TH. The transducer is displaced by 0.5 mm in all six directions: (c) +S, (d) +I, (e) +A, (f) +P, (g) +R, and (h) +L, relative to its base location. The blue lines denote the half-maximum pressure profiles in free water space.

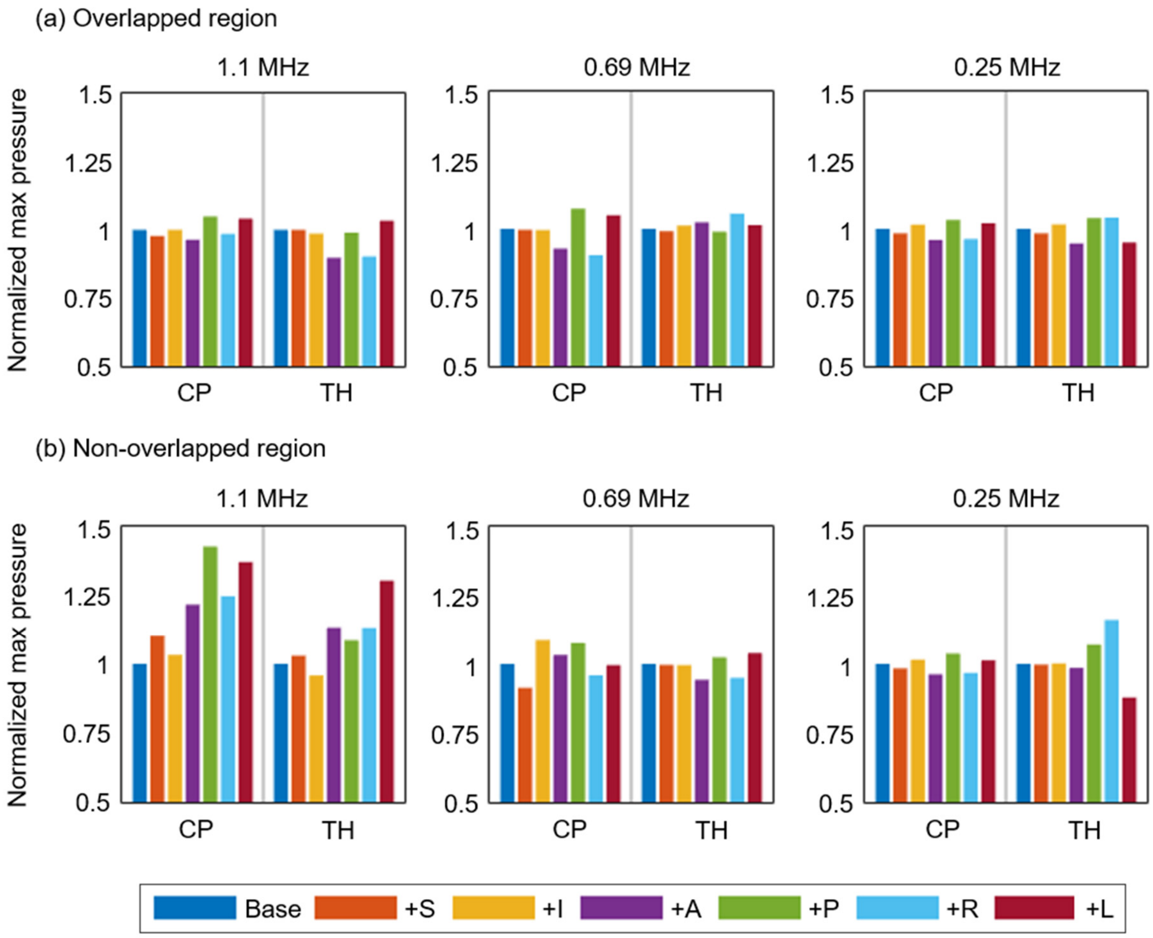

Supplementary Figure S2. Effects of transducer displacement on normalized intracranial maximum pressure in the overlapped (a) or non-overlapped (b) focal volume compared with the base model at 1.1, 0.69, and 0.25 MHz. The transducer was displaced by 0.5 mm in all six directions, +S, +I, +A, +P, +L, and +R, relative to its base location.
